# RNA-As-Graphs Motif Atlas—Dual Graph Library of RNA Modules and Viral Frameshifting-Element Applications

**DOI:** 10.3390/ijms23169249

**Published:** 2022-08-17

**Authors:** Qiyao Zhu, Louis Petingi, Tamar Schlick

**Affiliations:** 1 Courant Institute of Mathematical Sciences, New York University, 251 Mercer St., New York, NY 10012, USA; 2 Department of Computer Science, College of Staten Island, City University of New York, 2800 Victory Blvd., Staten Island, NY 10314, USA; 3 Department of Chemistry, New York University, 100 Washington Square East, New York, NY 10003, USA; 4 NYU-ECNU Center for Computational Chemistry, NYU Shanghai, Shanghai 200062, China; 5 NYU Simons Center for Computational Physical Chemistry, New York University, 24 Waverly Place, New York, NY 10003, USA

**Keywords:** coarse-grained RNA motifs, dual graphs and subgraphs, viral frameshifting elements, riboswitch structures

## Abstract

RNA motif classification is important for understanding structure/function connections and building phylogenetic relationships. Using our coarse-grained RNA-As-Graphs (RAG) representations, we identify recurrent dual graph motifs in experimentally solved RNA structures based on an improved search algorithm that finds and ranks independent RNA substructures. Our expanded list of 183 existing dual graph motifs reveals five common motifs found in transfer RNA, riboswitch, and ribosomal 5S RNA components. Moreover, we identify three motifs for available viral frameshifting RNA elements, suggesting a correlation between viral structural complexity and frameshifting efficiency. We further partition the RNA substructures into 1844 distinct submotifs, with pseudoknots and junctions retained intact. Common modules are internal loops and three-way junctions, and three submotifs are associated with riboswitches that bind nucleotides, ions, and signaling molecules. Together, our library of existing RNA motifs and submotifs adds to the growing universe of RNA modules, and provides a resource of structures and substructures for novel RNA design.

## 1. Introduction

As the versatile roles of RNA in gene editing and regulation have become known, RNA-based therapeutics has become an important application. For example, the discovery of the RNA interference (RNAi) pathway in the 1990s led to post-transcriptional gene expression regulation using microRNA or small interfering RNA [1,2], and clinical progress has followed (e.g., *patisrian* for treating transthyretin amyloidosis [3]). Similar ideas apply to anti-sense oligonucleotides [4,5,6]. With the emergence of CRISPR-Cas9 gene editing technology to directly knock out a gene at the DNA level, many applications are now possible to treat sickle cell anemia, HIV, and cancer [7]. In addition, RNA aptamers that bind to proteins expand the targeting cellular regions to extracellular spaces, which facilitates drug delivery [8]. Undoubtedly, RNA-based therapy has tremendous potential for addressing human disease, including virus infections, as evident in the success of the COVID-19 mRNA-based vaccines.

To accomplish these scientific achievements, knowing the target RNA structure is essential. Unlike DNA, which forms stable double helices, RNA is a flexible single strand that folds upon itself using Watson–Crick base pairs (A-U and G-C) and wobble base pair (G-U), and can thus form many complex structures in three dimensions (3D). Consecutive base pairs define *stems*, while residues without A-U, G-C, or G-U pairing form different types of *loops*, including *bulges*, *internal loops*, *hairpins*, and *junction loops*.

Coarse-grained models, especially graphs, have long been used to represent these RNA structures, since the pioneering works of Waterman [9], Shapiro [10], Nussinov [11], and others. Notably, in 1978, Waterman and Smith proposed graph representations of RNA 2D structures, where RNA residues are denoted as vertices [9]. In 1988, Shapiro proposed a tree graph representation, where RNA loops are denoted as vertices with their types labeled [10]. In 1989, Nussinov and coworkers introduced another tree graph representation, where both RNA stems and loops are denoted as vertices with their types and sizes labeled [11].

**Figure 1:**
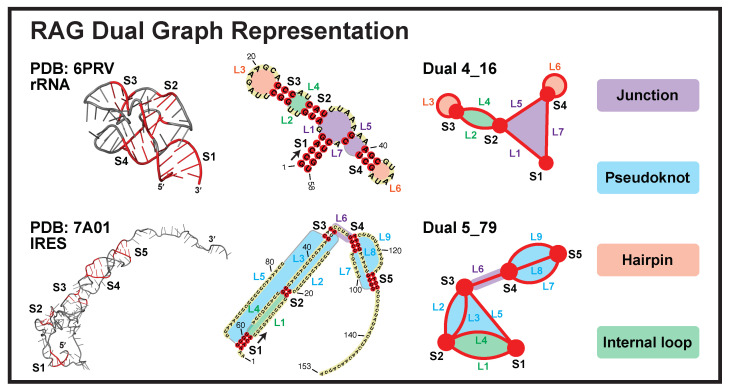
Dual graph representations for a 23S rRNA fragment (PDB ID: 6PRV) and an internal ribosomal entry site (IRES, PDB ID: 7A01). The stems are colored red and loops grey in the 3D structures. The different loops are labeled in the 2D structures and dual graphs. For the 2D structures (middle), an arrow is drawn at the 5${}^{\prime }$ end to show the sequence direction, and some residue numbers are given.

In 2003, our group launched the “RNA-As-Graphs” (RAG) framework with both tree and dual graph representations [12]. Our simplified tree graphs represent RNA loops as unlabeled vertices, and stems as connecting edges. Later, 3D tree graphs were defined for RNA tertiary structures, with additional vertices introduced for stem ends and small internal loops (<2-nt in either strand), as well as edges scaled according to stem lengths [13]. For dual graphs, we reverse the definitions so that stems are vertices and loops are edges (Figure 1). In this way, dual graphs can represent RNA pseudoknots (binding of a hairpin/bulge/internal loop to a single-stranded region outside of the helix).

Throughout these developments, our RAG toolkit has expanded to help define a motif atlas and design RNA motifs (see Table 1). Importantly, the mathematical enumeration of graphs allowed us to present an atlas of 2288 tree graphs of 1–13 vertices and 110,668 dual graphs of 1–9 vertices [12,14]. Among these, only a small portion correspond to real RNA molecules discovered, and we call these “existing”. The remaining graphs are “hypothetical”, and can be further divided into “RNA-like” and “non RNA-like”, by graph feature selection and clustering [15,16]. The “RNA-like” graphs are more likely to be found in nature, as our studies have shown (see also later in this manuscript) [15]. Hence, they are ideal candidates for novel RNA motif design, using our pipeline [17] that combines graph partitioning [18,19], fragment assembly [20], and inverse folding [21]. Indeed, our 3D substructure libraries RAG-3D and RAG-3Dual, which contain atomic fragments extracted from available RNA molecules [14,22], are important for finding similar RNA structures and designing novel motifs. Besides these graph motif libraries and RNA design initiatives, we have recently applied RAG tools to the SARS-CoV-2 frameshifting element (FSE), to define its complex conformational landscape and propose new anti-viral strategies based on mutations and conformational flexibility [23,24,25].

In this work, we present an updated RNA motif atlas for “existing” dual graphs (last reported in 2019 [14]), with corresponding subgraphs, using a new search algorithm defined to separate independent substructures in RNA molecules from the Protein Data Bank (PDB). Importantly, pseudoknots in the substructures are retained intact. With this new search algorithm, we match more dual graphs to existing RNAs. In particular, all 10 RNA-like (hypothetical) candidates we predicted in 2004 [28] are now found in Nature. The five top motifs occur in tRNA, nucleotide riboswitch, and ribosomal 5S RNA molecules. In the corresponding library of dual subgraphs obtained by partitioning of dual graphs [19], we identify many interesting submotifs in large ribosomal RNAs. Finally, we report on two applications to viral frameshifting elements (FSEs) and riboswitches. By collecting available FSEs, we observe relationships between motifs and phylogeny, as well as correlations between motif complexity and frameshifting efficiency. For riboswitches, we identify submotifs specific to certain riboswitch types, which may help identify new family members. Overall, our dual graph motif and submotif library offer a resource for identifying and searching biologically important RNA motifs.

## 2. Results

### 2.1. RNA Database

To identify existing dual graphs, we use the representative set defined by Bowling Green State University (BGSU) [29], which contains 2777 non-redundant RNA molecules from the PDB. Comparing to the 2019 dual graph library [14], 683 new RNA molecules are included. After extracting 2D structures using 3DNA-DSSR [30], there are two synthetic RNA molecules (PDB ID: 7BPG and 7BPF) that have unusual base pairs to be recognized, and are thus removed from the list (see Supplementary Figure S1).

### 2.2. Round 1: Substructure Search

**Figure 2:**
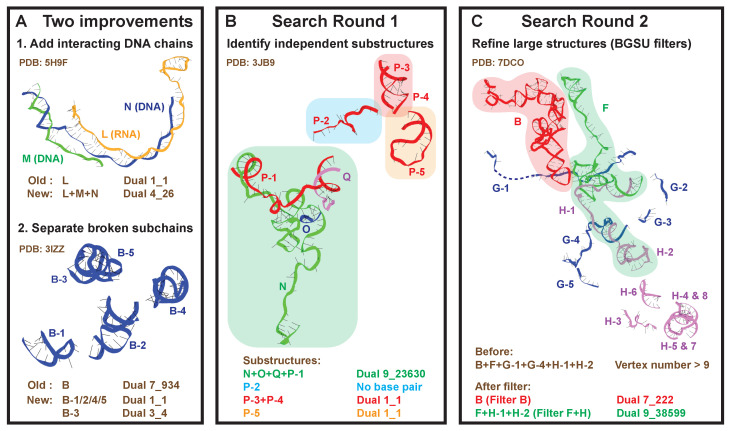
New search strategy to identify existing dual graphs. (**A**) As shown by two examples, we now include DNA chains that have strong interactions with RNA chains, and separate broken subchains. (**B**) The first round of the search algorithm groups all interacting subchains to find independent substructures and assigns corresponding dual graphs. (**C**) The second round refines large substructures with >9 helices using BGSU representative chains as filters.

We develop a new search algorithm to identify all independently folded nucleic acid substructures within these 2775 PDB molecules. Previously, coupled RNA chains were extracted after optimizing factors such as resolution and steric clashes [14,29]. However, there are two major problems with this approach, as shown in Figure 2A: (1) some RNA chains have strong interactions with DNA chains; and (2) some chains contain discontinuous subchains due to experimental resolution issues.

To solve these problems, we now consider all nucleic acid chains and work on the level of subchains. Indeed, we group subchains that interact with each other to define independent substructures and assign corresponding dual graphs (see details in Materials and Methods). We also record whether a substructure is made of a single continuous subchain, or multiple subchains, and whether DNA is involved.

For illustration, we show the analysis of a yeast spliceosome (PDB ID: 3JB9, Figure 2B). It contains four intertwining RNA chains (N, O, Q, P), and chain P is composed of five subchains. Our new search algorithm finds that chain N interacts with P-1 and O, chain P-1 interacts with Q and N, while chain O only interacts with N, and chain Q only with P-1. Together, chains N, O, Q, and P-1 form a substructure with dual graph 9_23630. Likewise, we find a helix formed by P-3 and P-4 (dual 1_1), a helix by P-5 alone (dual 1_1), and a single-stranded P-2.

**Figure 3:**
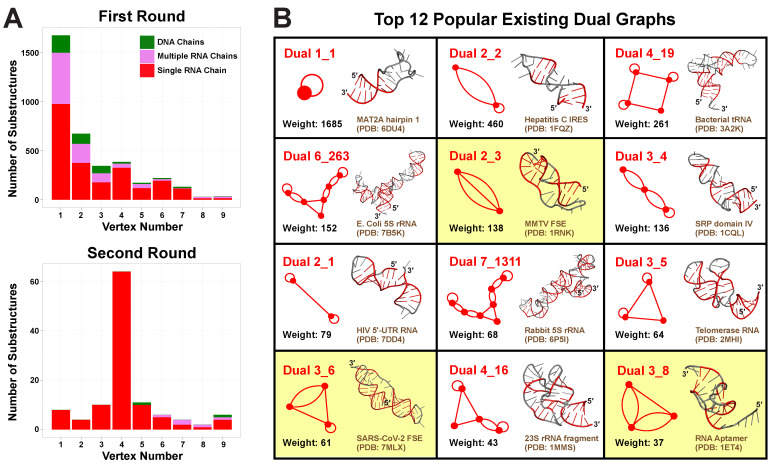
Existing dual graph distribution analysis. (**A**) Substructure distributions over dual graph vertex number. For each vertex number 1≤n≤9, substructures corresponding to dual graphs of *n* vertices found in the first and second search round are counted and separated into three cases: single RNA chain, multiple RNA chains, or DNA containing. (**B**) Top 12 popular existing dual graphs with their weights and example 3D structures. Graphs containing pseudoknots are highlighted in yellow.

After applying the first search round, we identify 28,336 substructures from the non-redundant set of 2775 PDB molecules. Using our dual graph representation, where RNA stems are denoted as vertices and loops as edges (see Figure 1 and detailed definitions in Materials and Methods), 1677 substructures are assigned to dual graph 1_1, and 1997 substructures are assigned to 170 unique dual graphs with 2–9 vertices (Figure 3A). For the remaining substructures: 23,996 have no helices (≤1 base pair) and 665 have >9 helices; 1 substructure has 8 helices but no matching graph (see Supplementary Figure S1), which indicates that our dual graph enumeration is imperfect, as expected from a heuristic enumeration process [14].

A clear decreasing trend is observed for the number of substructures as graph vertex number increases, so most substructures are small RNAs of 1–4 helices (Figure 3A). Nevertheless, the number of distinct existing graphs increases with vertex number, until reaching a maximum of 34 at vertex number 6 (Supplementary Table S1). Interestingly, the percentage of substructures made of single continuous RNA chains also peaks at vertex number 6, corresponding to 88.3%. This suggests that 6 helices are optimal for RNA motif variability, in the sense that this size is not too small to display variety, yet not too large for a single RNA strand to fold onto.

### 2.3. Round 2: Add Refinement for Large Substructures

For the 665 large substructures with >9 helices, instead of discarding them, we extract meaningful blocks that correspond to catalogued dual graphs. We use the representative chains from the BGSU list as “filters”, which group major chains with persistent base pairs [29]. For a large substructure, we take each BGSU representative in turn to identify subchains contained in it, and group them into independent blocks like before.

For example, the Cryo-EM structure of an activated human spliceosome (PDB ID: 7DCO, Figure 2C) contains chains B, F, G, and H. A large substructure consisting of (sub)chains B, F, G-1, G-4, H-1, and H-2 has more than 9 helices, and hence no dual graph assigned. Using BGSU representative chain B as the first “filter”, we identify chain B from the original large substructure, and we assign dual graph 7_222 to it. Using representative chain F+H as the second “filter”, we identify subchains F, H-1, and H-2, and they interact with each other to form dual graph 9_38599.

After this refinement, we filter out 324 substructures, and 115 of them are assigned to 35 unique dual graphs, including 11 new motifs from the first round (Figure 3A, Supplementary Table S1). This time, we find 93.9% of the 115 substructures correspond to continuous single RNA chains, mainly because the filters we use are mostly single chains. The majority of the substructures correspond to dual graphs of 4 vertices, again suggesting prevalence of small RNAs.

### 2.4. Popular RNA Motifs

We list 12 most popular motifs in Figure 3B with representative RNA structures. Ten of these popular motifs correspond to small RNAs of 1–4 helices. Motif 1_1 has the largest weight 1685 (number of corresponding RNA structures), which represents a single hairpin. Since we are using non-redundant RNA structure database, this weight is an approximate measure of structural variation of a given dual graph motif, and likely reflects the distribution of different RNA motifs in Nature. For example, the 1_1 hairpin forms in numerous contexts of sequences and stem/loop sizes. The next popular motif 2_2 represents two stems connected by an internal loop, and it is found in many RNAs, including the internal ribosome entry site for Hepatitis C virus (PDB ID: 1FQZ).

**Figure 4:**
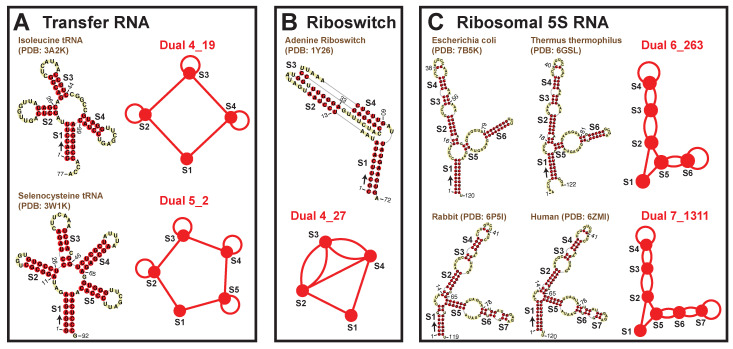
RNA functional classes associated with existing dual graph motifs. Motifs 4_19 and 5_2 are specific to tRNAs, motif 4_27 is specific to nucleotide riboswitches, and motifs 6_263 and 7_1311 is specific to 5S rRNAs. In each 2D structure, an arrow is drawn at the 5${}^{\prime }$ end to show the sequence direction, and residue numbers are labeled.

Among the popular motifs (weights ≥ 20), we find five that correspond to certain RNA classes: tRNAs, riboswitches, and ribosomal 5S RNAs (Figure 4). A four-way junction 4_19 and a five-way junction 5_2 correspond to tRNAs. Motif 4_19 has weight 261 and represents most tRNAs, including those carrying anticodons for Proline, Tryptophan, Methionine, Alanine, Asparagine, etc. Motif 5_2 (weight 36) has an extra stem (S4) that is called the *variable arm* [31], and it only represents tRNAs for Leucine, Tyrosine, and Selenocysteine. Besides these, pseudoknotted motif 4_27 (weight 30) is specific to riboswitches that bind nucleotide derivatives. Two other interesting motifs are 6_263 (weight 152) and 7_1311 (weight 68), both corresponding to ribosomal 5S RNAs. The 5S rRNA of length ∼120-nt has three helical arms, containing 1, 3, and 2 or 3 stems, respectively. Motif 6_263 is actually a subgraph of 7_1311, and typical examples are rRNAs from bacteria such as *E. coli* and *Thermus thermophilus*. For 7_1311, two example rRNAs are from rabbit and human.

### 2.5. Updated Existing Dual Graph Library

Comparing to the previous existing dual graphs, we now have 182 existing graphs versus 122 before 2019 [14], for dual graphs up to 9 vertices, and 86 are common (Supplementary Table S1). When checking the 36 graphs absent in the current list, we find most included in other ways, as follows (Supplementary Table S2). Graph 5_6 with highest weight corresponds to 4 CRISPR-Cas9 complexes, and using our new search algorithm, DNA chains are included and the four complexes are assigned to larger graphs of 6 to 7 vertices. Similarly, 15 previous graphs (weights 1–2) which contain broken chains are included as smaller substructures. Another 10 previous graphs (weights 1) did not include interacting chains. The final 10 graphs (weights 1) corresponded to different 2D structures in our prior study due to different 2D extraction procedures used (see details in Supplementary Information and Supplementary Figure S2) [14]. For these 10 structures, we perform additional screening using two other 2D extraction programs RNAView [32] and MC-Annotate [33]. We find seven of them have consistent motifs with our current results, i.e., at least two of three programs produce the same motif as we identify here, and three have consistent motifs with the prior study (Supplementary Table S3). Hence, we re-assign these 3 structures with the prior motifs (PDB ID: 6D9J, 5XY3, 5IT9). Clearly, small differences in search algorithms induce variations in resulting motifs, but the motif library is generally robust.

It is also interesting to examine in this light the 96 newly found existing dual graphs (Supplementary Table S4). There are 40 graphs (of weights 1–3) that correspond to newly solved RNA structures since our last update in August 2018 [14]. Another 21 graphs (weights 1–9) represent RNA-DNA hybrids; 7 graphs (weights 1–4) have RNAs with broken chains; and 23 graphs (weights 1–3 except for graph 5_43) correspond to RNAs with multiple interacting chains. Only five graphs are newly found due to 2D structure extraction differences, and two align with prior motifs after additional screening, including one found above (PDB ID: 6D9J, Supplementary Table S3). Interestingly, graph 3_7, which is the only 3-vertex graph not included in the previous existing dual graph list, is now identified with 9 structures, all containing DNA. This suggests that 3_7 is an uncommon motif for a single RNA chain. Indeed, this motif represents a flanked H-type pseudoknot, i.e., the two ends of a 2-stem H-type pseudoknot bind to form the third stem (Supplementary Figure S3). Overall, higher weighted graphs are common to both existing dual graph lists.

**Figure 5:**
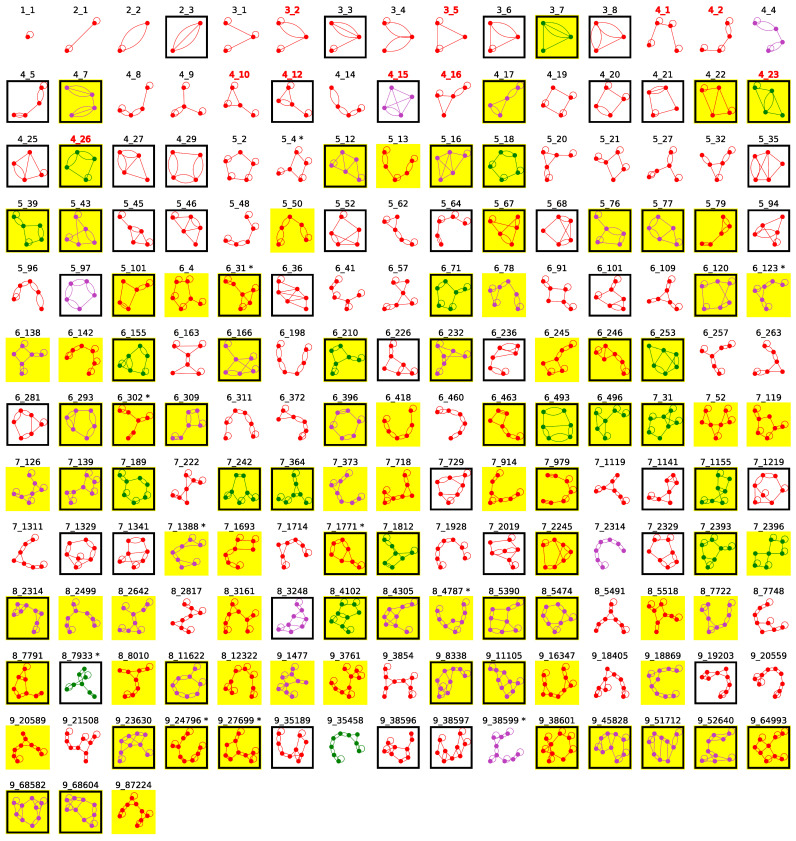
The current 183 existing dual graphs with 1–9 vertices. A graph is colored green if all substructures represented are RNA-DNA hybrids; otherwise, purple if all substructures consist of multiple RNA subchains; and red if at least one substructure has with a single RNA subchain. Graphs containing pseudoknots are boxed. Graphs found in our second search round are marked with asterisk superscript (after graph ID). Newly identified existing graphs are highlighted in yellow. The graph IDs of our 10 pseudoknot-containing RNA-like graphs proposed in 2004 are labeled red (top 3 rows) [28].

With slight 2D structure adjustments for 4 RNAs using additional screening (Supplementary Table S3), we now have 183 existing dual graphs (Table 2, Figure 5). About 50% of these graphs contain pseudoknots, regardless of the vertex number (boxed motifs in Figure 5), for a total of 100 pseudoknotted graphs.

Importantly, in this new set of existing dual graphs, all 10 initial RNA-like dual graph candidates we proposed and designed in 2004 are now “existing” [28]. Of these ten motifs, five (3_2, 3_5, 4_1, 4_2, and 4_16) were found in our 2011 study [34], three (4_10, 4_12, and 4_15) were added in our 2019 update [14] and, by considering RNA–DNA hybrids, we now found the last two candidates 4_23 (RNA polymerase elongation complex, PDB ID: 6FLQ) and 4_26 (CRISPR complex, PDB ID: 5H9F).

Besides our 2004 graph classification [28], we proposed an extended list of 78,742 RNA-like candidates out of the 110,667 enumerated dual graphs (2–9 vertices) in 2021, using Fiedler vector based graph feature selection and unsupervised K-means clustering (Table 1) [16]. Of this list, we find that 167 of the current 182 existing dual graphs (2–9 vertices) were indeed correctly classified as RNA-like (91.8% accuracy), and within the 94 newly found existing dual graphs, 85 were RNA-like (90.4%). The misclassified existing dual graphs are listed in Supplementary Table S5, and they are all large graphs (8–9 vertices) with small weights (≤5).

### 2.6. Subgraphs of Existing Dual Graph Motifs

**Figure 6:**
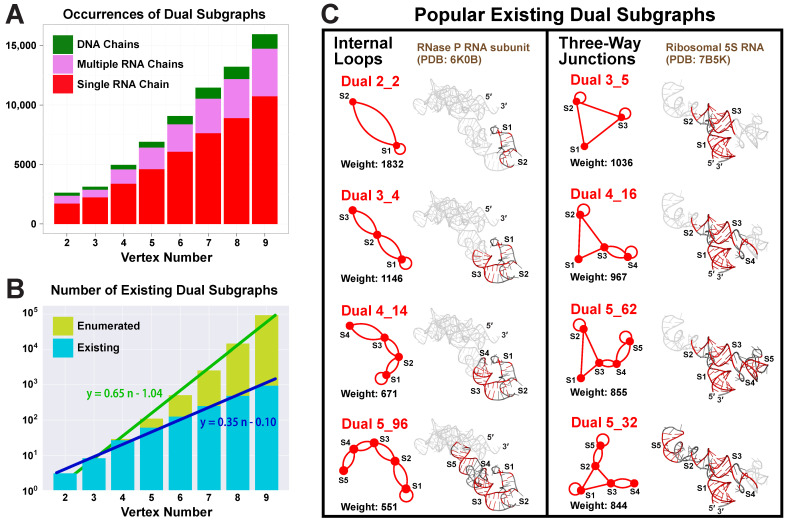
Dual subgraph distribution analysis. (**A**) For each vertex number 2≤n≤9, the occurrence of dual subgraphs of *n* vertices is counted. Those from substructures of single RNA chains, multiple RNA chains, and DNA chains are colored red, purple, and green, respectively. (**B**) Log plots of the number of existing dual subgraphs and the number of dual graphs enumerated over different vertex numbers, with linear least squares regressions performed. (**C**) Two groups of popular existing subgraphs with their weights. Sample RNAs are shown with submotif structures highlighted.

Our partitioning algorithm divides a dual graph into subgraphs while keeping pseudoknots and junctions intact (see details in Materials and Methods) [19]. Using this partitioning algorithm, we find 1844 distinct subgraphs of 2–9 vertices from the 2663 substructures that have ≥2 helices (no filters used). Unlike the one-to-one correspondence between a substructure and its dual graph, multiple subgraphs are contained in one substructure. As the vertex number increases, more subgraphs are found (Figure 6A).

Nevertheless, the subgraph compositions remain similar for all vertex numbers, with the majority (65–72%) coming from substructures of single RNA chains, some (20–26%) from those of multiple RNA chains, and few (7–9%) from those containing DNA chains. Considering that only 42.9% of the substructures are of single RNA chains, we see that these substructures contribute more subgraphs, mainly because there are many large ribosomal RNA chains. For example, a ribosomal 16S RNA (PDB ID: 4GKK) consisting of a single 1513-nt chain can be partitioned into 159 subgraphs of 2–9 vertices.

The number of distinct existing subgraphs increases exponentially with the vertex number *n*. By plotting the subgraph number in log scale, we see a linear relation with least squares regression y=0.35n−0.1, which corresponds to an original exponential relation of $y=0.79\cdot 2.24^{n}$ (Figure 6B). Similarly, the total number of enumerated dual graphs also has an exponential relation of $y=0.09\cdot 4.47^{n}$. Hence, the existing subgraphs follow the same type of exponential distribution as the total enumerated graphs, but with a slower rate.

### 2.7. Popular RNA Submotifs

The most popular subgraphs are of two types (Figure 6C). One type has stems connected by internal loops, including motif 2_2 (weight 1832), 3_4 (weight 1146), 4_14 (weight 671), and 5_96 (weight 551), with one stem added at a time by internal loops. Since smaller motifs are subgraphs of larger ones, they have higher weights. The RNA subunit of Ribonuclease P (PDB ID: 6K0B) contains all four subgraphs, and the submotifs are highlighted in its 3D structure. Another type of popular subgraphs contains three-way junctions. Starting from motif 3_5 (weight 1036), stems can be added to any of the three arms by internal loops. Motif 4_16 (weight 967) is obtained by adding S4 to arm S3, and 5_62 (weight 855) by further adding S5 to S4. Motif 5_32 (weight 844) adds a stem to both arms S2 and S3. These subgraphs are all contained in the ribosomal 5S RNA of *E coli.* (PDB ID: 7B5K).

**Figure 7:**
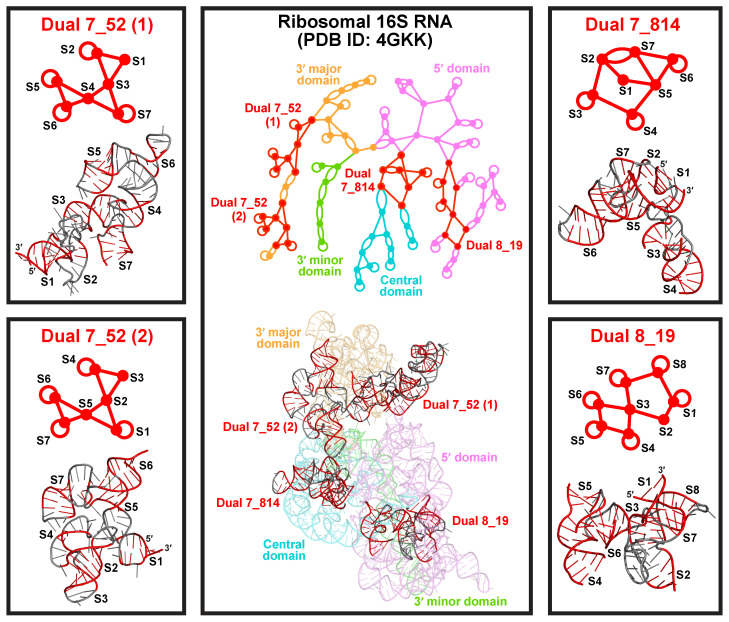
Three common motifs only form as subgraphs. Subgraph 7_52 appears in two different orientations in the 3${}^{\prime }$ major domain of *Thermus thermophilus* 16S rRNA (PDB ID: 4GKK). Subgraphs 7_814 and 8_19 appear in the central and the 5${}^{\prime }$ domain of the 16S rRNA, respectively.

All popular subgraphs mentioned above are also frequent in the existing dual graph list (weights ≥ 19), but there are also modules that only appear as subgraphs. These special modules mainly come from long rRNA chains. For example, the three large subgraphs 7_52 (weight 254), 7_814 (weight 168), and 8_19 (weight 309) are unique to the 16S and 18S rRNAs (Figure 7). Except for 7_52, which corresponds to two 18S rRNA substructures, these motifs do not form on their own as existing dual graphs, suggesting that they need to be stabilized by neighboring structures in large rRNAs.

Both motifs 7_52 and 8_19 are unknotted composites of junctions (Figure 7). Motif 7_52 consists of 3 three-way junctions, and appears twice in the 3${}^{\prime }$ major domain of *Thermus thermophilus* 16S rRNA (PDB ID: 4GKK). The two occurrences have different orientations, in the ordering of helices from the 5${}^{\prime }$ to 3${}^{\prime }$ end. While the first 7_52 RNA fragment is longer and more elongated, the overall structures are very similar. Motif 8_19 consists of a four-way and a five-way junction, and appears in the 5${}^{\prime }$ domain. Because of these junction composites, these RNA structures can have many branches and are more compact.

Motif 7_814 contains two intertwined three-way junctions and a flanking stem (Figure 7). In it, Stems 2, 3, and 4 form one junction, and Stems 5, 6, and 7 form another junction, while Stems 2 and 7 intertwine to form a pseudoknot. Stem 1 encompasses the whole structure by joining the 5${}^{\prime }$ and 3${}^{\prime }$ ends. This complex structure exists in the central domain of the 16S rRNA. As we see, the different rRNA domains have their own favorable motifs, which probably helps serve biological functions.

### 2.8. Applications: Delineating Frameshifting Element and Riboswitch Motifs

Our updated library of existing dual graphs and their subgraphs allows us to easily identify representative motifs for functional RNA groups. Because these RNAs perform regulatory roles by binding to proteins, nucleic acids, or ions, their structures are often conserved, and cataloging their motifs helps understand associated mechanisms, trace evolutionary relationships, and discover new members. Here, we illustrate these ideas for viral frameshifting elements (FSE) and riboswitches.

**Figure 8:**
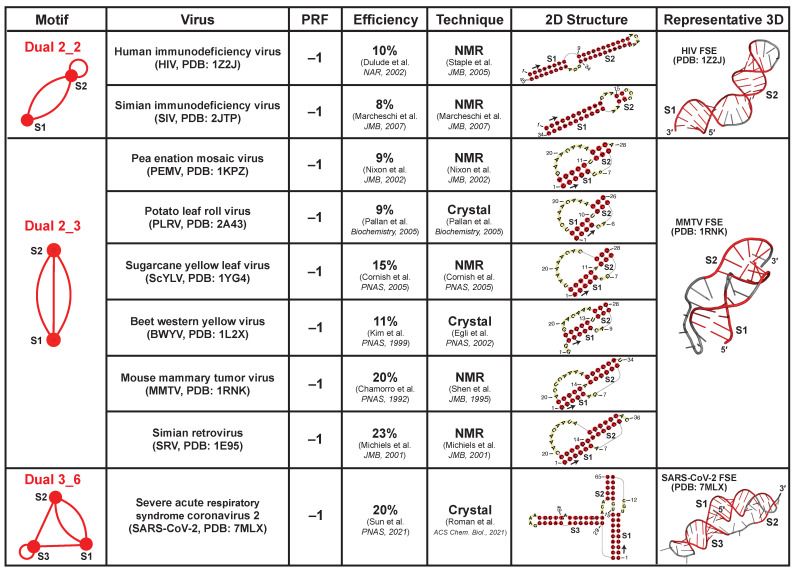
Experimentally solved frameshifting element structures available in the Protein Data Bank [40,41,42,43,44,45,46,47,48]. The 9 FSEs are grouped by their dual graph motifs. They all promote −1 programmed ribosomal frameshifting (PRF). The in vitro frameshifting efficiencies in literature are provided [41,42,43,44,47,49,50,51,52]. For each extracted 2D structure, an arrow is drawn at the 5${}^{\prime }$ end to show the sequence direction, and some residue numbers are labeled.

Viral frameshifting elements (FSEs) are small mRNA regions (<100-nt) that stall the ribosome and shift the reading frame to ensure correct translation of overlapped open reading frames. This strategy is commonly used by viruses like coronaviruses, and it is believed that the FSE RNA structure is essential for triggering frameshifting [24,35,36]. Here, we collect all nine available FSEs in the PDB and identify three dual graph motifs (Figure 8): dual graph 2_2 for FSEs of Human immunodeficiency virus (PDB ID: 1Z2J) and Simian immunodeficiency virus (2JTP); 2_3 for FSEs of Sugarcane yellow leaf virus (1YG4), Beet western yellow virus (1L2X), Pea enation mosaic virus (1KPZ), Potato leaf roll virus (2A43), Simian retrovirus type-1 (1E95), and Mouse mammary tumor virus (1RNK); 3_6 for FSE of Severe acute respiratory syndrome coronavirus 2 (7MLX). These FSEs all promote −1 ribosomal frameshifting, i.e., the ribosome backtracks 1-nt to resume protein translation from the RNA transcript. Their dual graphs are all non-separable and cannot be partitioned.

These FSEs either form long stems like the 2_2 motif, or intertwined pseudoknots like the 2_3 and 3_6 motifs (the latter is the SARS-CoV-2 FSE). These structures are difficult to unwind, and hence could provide mechanical barrier to stall the ribosome and facilitate frameshifting [37,38]. Moreover, the pseudoknot motifs typically have higher frameshifting efficiencies (9–23%) compared to the unknotted 2_2 motif (8–10%), probably due to the formation of the intertwined topology as well as stem–loop/loop–loop interactions [39].

We also identify a phylogenetic relation between the motifs and the viruses. The 2_2 motif corresponds to HIV and SIV FSEs. Both viruses belong to the *Lentivirus* genus, and they are closely related in evolution [53]. The 2_3 pseudoknot is the most popular motif. Interestingly, all plant virus FSEs (*Solemoviridae* family) have this motif. Thus, using our dual graph motif classification, we can identify and group similar RNAs to suggest phylogenetic connections among organisms.

**Figure 9:**
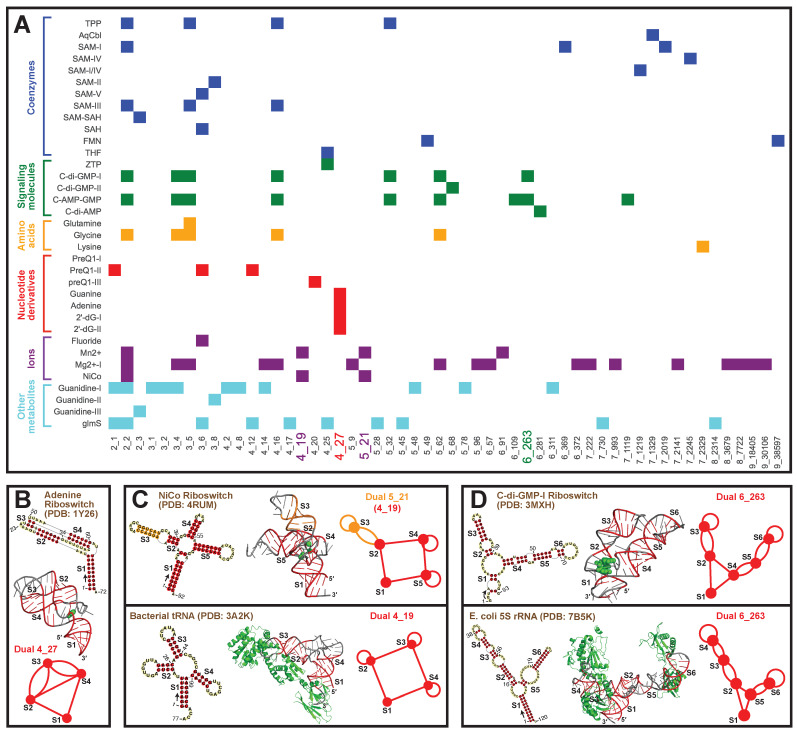
Graph motifs for riboswitches in the Protein Data Bank. (**A**) Motif distributions of the 35 riboswitches are shown as heatmap. The riboswitches are classified into 6 groups based on their ligands on the *y*-axis, and colored differently. All subgraphs found are listed on the *x*-axis. (**B**) Motif 4_27 is unique to nucleotide riboswitches, and the adenine riboswitch (PDB ID: 1Y26) is shown, with ligand in green. For each extracted 2D structure, an arrow is drawn at the 5${}^{\prime }$ end to show the sequence direction, and some residue numbers are noted. (**C**) Motif 5_21 (subgraph 4_19) is unique to ion riboswitches, with example of NiCo riboswitch (PDB ID: 4RUM). A comparison with bacterial tRNA (PDB ID: 3A2K) of motif 4_19 is shown below. (**D**) Motif 6_263 is unique to signaling molecule riboswitches, with example of C-di-GMP-I riboswitch (PDB ID: 3MXH). A comparison with *E. coli* 5S rRNA (PDB ID: 7B5K) of motif 6_263 is shown below.

Similarly, for riboswitches, which are RNAs that bind ligands and alter their structures to regulate gene expression, we classify the 35 riboswitch types available in PDB into six groups based on their ligands (coenzymes, signaling molecules, amino acids, nucleotide derivatives, ions, and other metabolites), following [54]. Corresponding dual graphs are identified for the 2D structures and partitioned into subgraphs. A total of 54 unique subgraphs are found, with relevant riboswitches shown as a heatmap in Figure 9A. We see that subgraph 2_2 is most popular and is a component of all riboswitches except those that bind to nucleotide derivatives.

Motif 4_27 is only seen in nucleotide riboswitches. Indeed, of all PDB structures, this motif is specific to these riboswitches. This specificity could then be used to find novel nucleotide riboswitches. An illustrative nucleotide riboswitch that binds to adenine (PDB ID: 1Y26) is shown in Figure 9B. It contains a 3-stem kissing-loop pseudoknot, i.e., the loop regions of Stems 2 and 4 base pair to form Stem 3, and the 5${}^{\prime }$ and 3${}^{\prime }$ ends base pair to form flanking Stem 1. The ligand binds to the junction between Stem 1 and the pseudoknot.

Two other interesting motifs are 5_21 and 6_263, both unknotted. Motif 5_21 is found in ion riboswitches, and it contains a four-way junction (subgraph 4_19). Among all PDB structures, this 4_19 junction is also seen in tRNAs. A comparison between NiCo riboswitch (PDB ID: 4RUM) and bacterial tRNA (PDB ID: 3A2K) is shown in Figure 9C. An additional Stem 3 (orange) extends one of the helical arms Stem 2 in the riboswitch, while the rest of the junction looks similar to the L-shape of tRNA. The binding pockets are different though: in the riboswitch, the ion binds to the stem junction; in the tRNA, the proteins bind to the stem loops. Likewise, motif 6_263 exists in signaling molecule riboswitches and 5S rRNAs (Figure 9D). The molecule binds to the junction region in the riboswitch, while the proteins bind to the stem loops in the rRNA.

## 3. Discussion

Using dual graph representations that can represent RNA pseudoknots, we have annotated the dual graph motif atlas up to 9 vertices to identify all existing RNA motifs and described their subgraphs. We improved our search algorithm to identify independently folded RNA substructures in the PDB by including interacting DNA chains and separating broken chains. The result is a list of 183 existing dual graphs of 1–9 vertices, containing all 10 RNA-like candidates we predicted in 2004 [28]. The popular dual graph motifs include five that correspond to certain RNA families: junction 4_19 and 5_2 for tRNAs, pseudoknot 4_27 for riboswitches, and 3-helical-arm 6_263 and 7_1311 for 5S rRNAs. The partitioned 1844 subgraphs include many new motifs arise from long rRNA chains, including compact junction composites.

As an application of this RNA motif catalog, we have classified all available viral frameshifting elements in the PDB into three groups: unknotted 2_2, and pseudoknotted 2_3 and 3_6, and noted higher frameshifting efficiencies for the pseudoknots. For riboswitches, motifs specific to certain types of riboswitches were identified, such as 4_27 for nucleotide riboswitches and 5_21 for ion riboswitches. From both applications, we see how dual graph classification and partitioning can help catalog and analyze common motifs for functional RNAs. These common motifs not only suggest phylogenetic relations among different organisms, but also help in structure/function connections. Relating the motifs to important biological features such as the frameshifting efficiency can lead to enhanced understanding of the associated mechanisms. The application of relating frameshifting to energy landscape of coronaviruses is underway [27].

Our dual graph atlas is complementary to other RNA databases. Besides the PDB, there are databases that collect certain types of RNAs, such as *Rfam* for non-coding RNAs and cis-regulatory elements [55], *UTRdb* for untranslated eukaryotic mRNA regions [56], *ASD* for alternative splicing sites [57], and *TRANSFAC* for transcription factors [58]. Similar to the FSE application, we can classify dual graph motifs in these RNA families. Compared to the traditional consensus 2D structure construction using multiple sequence alignment and covariance models, our coarse-grained dual graph representation can quickly group similar RNA structures due to invariance to stem and loop sizes.

Like other databases, our dual graph atlas helps future motif search. Functional RNAs often rely on their structures to accomplish biological roles, such as binding proteins. For a novel RNA molecule, finding known RNAs that have similar structures can help decipher its function. Hence, using our dual atlas as an annotation and query tool, we can investigate other RNAs that have the same dual graph representation. Our RAG-3Dual database, which records available RNA fragments, can be further used to find 3D RNA structures and substructures that have these dual graph and subgraph representations [14].

Another advantage of our dual graph representation is that we can partition the RNAs into biologically meaningful blocks. Specifically, we maintain junctions and pseudoknots intact. The freedom of combining adjacent blocks at different articulation points allows different levels of division. Since RNAs are modular, finding popular building blocks is important for RNA structural biology [59], especially for large RNA molecules such as ribosomal RNAs. Moreover, as functional RNAs often have conserved structures, differences within their substructure blocks can provide clues on evolutionary processes. In our prior study, we have deduced ancestry relationships of different rRNAs using subgraph block distributions [60].

In summary, our dual graph representation provides a quick and alternative way to collect and classify RNA motifs. Applications to functional RNAs can help trace family evolutions and interpret biological mechanisms. Future development of 3D dual graphs to compare and search similar RNA substructures containing pseudoknots can be fruitful, following similar protocols of 3D tree graphs [22]. Building a subgraph library would complement our motif atlas, enhance our understanding of common RNA blocks, and serve as components for novel RNA design.

## 4. Materials and Methods

### 4.1. Dual Graph Definitions

Our dual graphs are defined by denoting RNA stems as vertices, and loops as edges. The exact representation rules are as follows (two examples shown in Figure 1):

- Each junction loop is denoted as an edge (e.g., purple loops in Figure 1).
- Each single strand in a pseudoknot is denoted as an edge (e.g., blue loops).
- Hairpin loops are denoted as self-edges (e.g., orange loops).
- A single-stranded internal loop/bulge is denoted as two edges (e.g., green loops). A bulge of 1-nt or an internal loop of 1-nt on both sides is ignored.
- A stem of ≥2 consecutive base pairs (i.e., no loop in between) is denoted as a vertex. An isolated single base pair is ignored.
- The dangling 5${}^{\prime }$ and 3${}^{\prime }$ ends are ignored.

### 4.2. RAG Library

Our RAG libraries are available on http://www.biomath.nyu.edu/?q=rag/home (accessed on 1 August 2022). In our prior study, we have enumerated by a heuristic method 110,668 dual graphs of 1–9 vertices by iteratively connecting small graphs [14]. Dual graphs of the same number of vertices *V* are sorted in ascending order of their Fiedler values and assigned with IDs V_n. As Fiedler values reflect the connectivity/compactness of the graphs [61], graphs with larger *n* are more connected/compact. Among all graphs enumerated, those corresponding to RNAs found in Nature are termed “existing”, and the number of corresponding structures are assigned as “weights”.

### 4.3. New Substructure Search Algorithm

In our new search algorithm, we identify independently folded nucleic acid substructures with two improvements: (1) include DNA chains that have strong interactions with RNA chains; and (2) separate broken subchains. For example, in a CRISPR system (PDB ID: 5H9F, Figure 2A), a single-stranded CRISPR RNA (chain L) binds to a target DNA strand (chain N) while replacing its complementary DNA strand (chain M). The previous algorithm only accepted the RNA chain L and assigned it dual graph 1_1. Nevertheless, the folding of chain L could be completely different without chains N and M. Therefore, now we assign dual graph 4_26 to this RNA–DNA hybrid structure based on the combined chains L, M, and N.

An example for broken chains is the large ribosomal subunit of *E. coli* (PDB ID: 3IZZ, Figure 2A). There are five independently folded discontinuous subchains in chain B. The previous algorithm did not notice this and assigned dual graph 7_934 to the entire chain B. Here, we identify and number these five subchains as B-1 to B-5, and assign individual dual graphs to each (four 1_1 graphs and one 3_4). This assignment is biologically meaningful, as the five subchains correspond to five ribosomal RNA helices in the original experiment [62]. Below is the exact search algorithm.

**RNA motif identification protocol:**

1. Identify the set of all subchains *C* using 3DNA-DSSR [30]. For each subchain *x*, find the set of subchains $Y_{x}$ that interacts with it (>1 base pairs). Subchains that have >92% sequence and 2D structure similarity to subchain *x* are not included in $Y_{x}$ to avoid polymers.
2. For each subchain *x* not assigned to any substructure, set its initial substructure $S_{x}=Y_{x}$, and add subchains that interact with any subchain in $S_{x}$. For those newly added subchains, again include their interacting subchains if not contained in $S_{x}$ yet. This process is repeated until no new subchains are added. Below is the corresponding pseudocode (Algorithm 1):

**Algorithm 1** Pseudocode for identifying independently folded nucleic acid substructures.**Input:** Set of all subchains *C*, Set of interacting subchains $Y_{x}$ for each subchain *x***Output:** Independently folded SubstructuresLibrary←*C*     ▹Library records all subchains not assigned to substructures**for***x* in *C* **do**    **if** *x* in Library **then**      ▹ Find the substructure containing subchain *x*        $S_{x}\leftarrow Y_{x}$        $S_{new}\leftarrow Y_{x}$      ▹$S_{new}$ records newly added subchains in each loop below        **while** $S_{new}$ not empty **do**           $S_{tmp}\leftarrow S_{x}$        ▹$S_{tmp}$ updates the substructure in each loop           **for** *z* in $S_{new}$ **do**               $S_{tmp}\leftarrow S_{tmp}\cup Y_{z}$  ▹ Include interacting subchains for each newly added subchain           **end for**           $S_{new}\leftarrow S_{tmp}\backslash S_{x}$           $S_{x}\leftarrow S_{tmp}$        **end while**        Library←$Library\backslash S_{x}$       ▹ Remove subchains in $S_{x}$ from Library        Add $S_{x}$ to Substructures    **end if**
**end for**



### 4.4. Subgraph Partitioning

**Figure 10:**
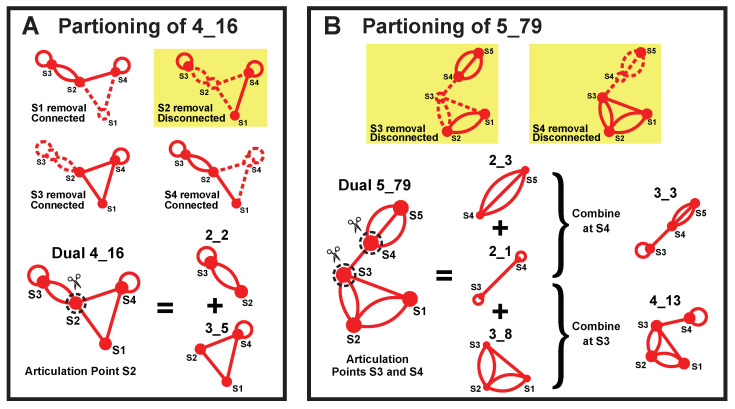
Dual graph partitioning. (**A**) For graph 4_16, articulation point S2 divides the graph into blocks (subgraphs) 2_2 and 3_5. (**B**) For graph 5_79, articulation points S3 and S4 separate the graph into blocks (subgraphs) 2_3, 2_1, and 3_8. Subgraphs 2_3 plus 2_1 correspond to 3_3, and subgraphs 2_1 plus 3_8 yield 4_13.

Our partitioning algorithm divides a dual graph into subgraphs while keeping pseudoknots and junctions intact [19]. The key is to identify “articulation points” in the dual graph. If the removal of an vertex and its incident edges results in disconnected graphs, that vertex is an articulation point. For example, in dual graph 4_16, vertex S2 is the only articulation point (Figure 10A). The articulation points separate the dual graph into maximal connected subgraphs called “blocks” [19]. As an example, for dual graph 4_16, we obtain blocks (subgraphs) 2_2 and 3_5.

If more than one articulation point exists, we can combine adjacent blocks (blocks that share an articulation point) to obtain more subgraphs. For example, dual graph 5_79 contains two articulation points S3 and S4, which separate the graph into three blocks (subgraphs) 2_3, 2_1, and 3_8 (Figure 10B). A combination of 2_3 and 2_1 at S4 yields subgraph 3_3, and a combination of adjacent blocks 2_1 and 3_8 at S3 produces 4_13.

## Figures and Tables

**Table 1:** RNA-As-Graphs (RAG) developments.

Year	RAG Development	Refs.
2003	Launch of RAG: planar tree and dual graphs	[12]
2011	RNA junction coaxial stacking prediction	[26]
2014	Tree graph partitioning using Fiedler vectors	[18]
2014	RAG 3D tree graph: sampling RNA 3D structures	[13]
2015	Laplacian spectrum based graph feature selection and clustering	[15]
2015	RAG-3D Database: searching for similar RNA fragments	[22]
2017	Fragment assembly (F-RAG): generating atomic models for tree graphs	[20]
2017	Dual graph partitioning algorithm	[19]
2018	Novel RNA motif design pipeline	[17]
2019	Extended dual graph library and RAG-3Dual database	[14]
2020	Tree graph inverse folding (RAG-IF)	[21]
2021	Fiedler vector based graph feature selection and scoring	[16]
2021	Dual graph inverse folding (Dual-RAG-IF) and	[23]
	SARS-CoV-2 frameshifting element (FSE) conformational landscape	[24]
2022	SARS-CoV-2 FSE dynamics and Coronavirus conformational landscape	[25,27]

**Table 2:** RNAs in Nature identified by dual graphs in our motif library. For each vertex, the number of total graphs enumerated are shown. For current existing graphs, those found in the first, the second, and the combined search round are counted against vertex number, as well as those contain pseudoknots. For comparison, the total number of existing dual graphs in the prior library [14] and those motifs common to both search protocols are listed.

Vertex	Graphs	Current Existing Graphs				Prior Existing
		Rd 1	Rd 2	Combined	Pknot	Total/(Common)
**1**	1	1	1	1	0	1 (1)
**2**	3	3	3	3	1	3 (3)
**3**	8	8	4	8	4	7 (7)
**4**	29	22	5	22	13	17 (17)
**5**	110	28	6	29	18	20 (17)
**6**	508	36	5	39	21	22 (16)
**7**	2551	31	4	33	18	21 (13)
**8**	14,670	18	3	20	9	14 (5)
**9**	92,788	25	4	28	16	17 (10)
**Total**	**110,668**	**172**	**35**	**183**	**100**	**122 (89)**

## Data Availability

The updated library of existing dual graphs and subgraphs are shared in the GitHub Schlicklab repository https://github.com/Schlicklab/Existing-Dual-Search (accessed on 1 August 2022). The codes for the dual graph motif search algorithm are also available in the repository.

## Supplementary Materials

The following supporting information can be downloaded at: https://www.mdpi.com/article/10.3390/ijms23169249/s1.
