# Supplementary material for: RNA-As-Graphs Motif Atlas—Dual Graph Library of RNA Modules and Viral Frameshifting-Element Applications
Source: Int J Mol Sci. 2022 Aug 17;23(16):9249. doi: 10.3390/ijms23169249 (PMC9408923; doi:10.3390/ijms23169249)
Supplement: Supplementary file 1 [file ijms-23-09249-s001.zip › ijms-1858762-supplementary.pdf]

Supplementary Information for *RNA-As-Graphs Motif Atlas — Dual Graph Library of RNA Modules and Viral Frameshifting-Element Applications*

Qiyao Zhu[1], Louis Petingi[2], and Tamar Schlick[1,3,4,5,*]

[1]Courant Institute of Mathematical Sciences, New York University, 251 Mercer St., New York, NY 10012 U.S.A.

[2]Department of Computer Science, College of Staten Island, City University of New York, 2800 Victory Blvd., Staten Island, NY 10314 U.S.A.

[3]Department of Chemistry, New York University, 100 Washington Square East, New York, NY 10003 U.S.A

[4]NYU-ECNU Center for Computational Chemistry, NYU Shanghai, Shanghai 200062, P.R. China

[5]NYU Simons Center for Computational Physical Chemistry, New York University, 24 Waverly Place, New York, NY 10003 U.S.A

[*]Corresponding author: schlick@nyu.edu

August 17, 2022

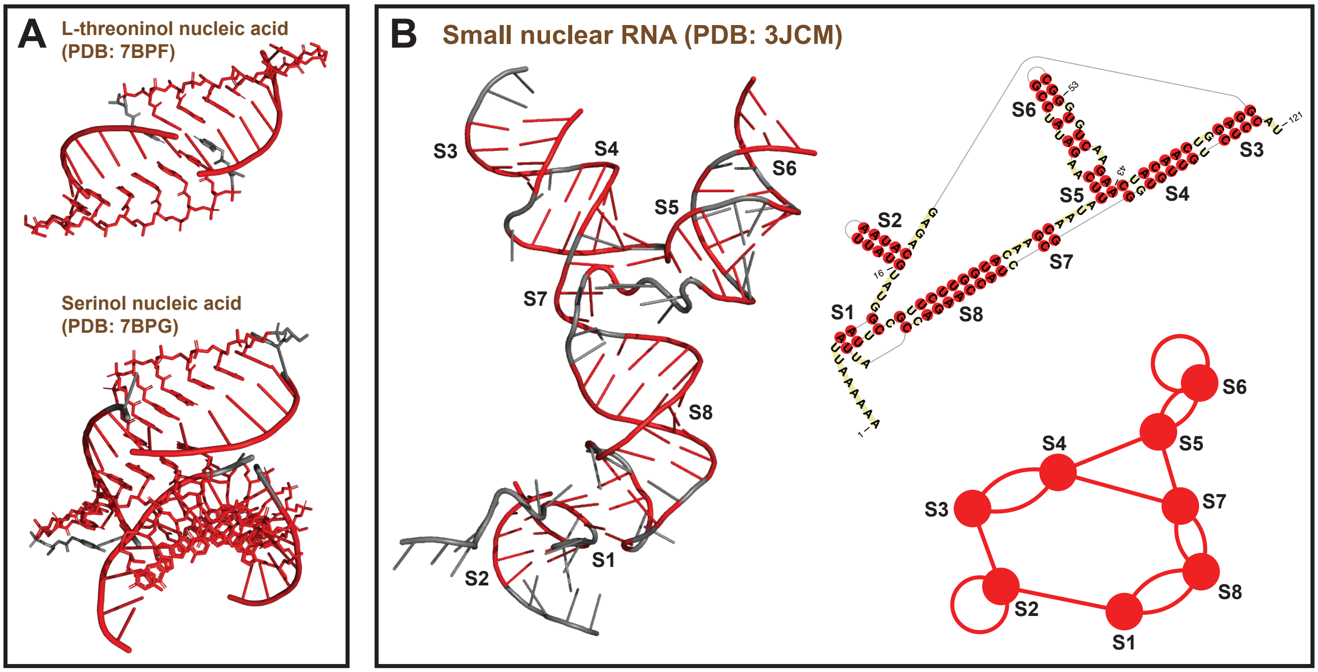

Figure S1: PDB RNA structures with no graphs assigned. (A) Two synthetic RNA molecules with strands formed by RNA residues (represented by smooth backbones and simplified nucleobase lines) and L-aTNA residues (acyclic$_L$-threoninol nucleic acid, represented by zigzag backbones and nucleobase rings). They have unusual 2D structures to be recognized. (B) A small nuclear RNA that has 8 helices but no matching graph in our dual graph library.

Vertex	Graphs	Current existing graphs				Prior existing
		Rd 1	Rd 2	Combined	Pknot	Total/(Common)
**1**	1	1	1	1	0	1 (1)
**2**	3	3	3	3	1	3 (3)
**3**	8	8	4	8	4	7 (7)
**4**	29	22	5	22	13	17 (17)
**5**	110	28	6	29	18	20 (17)
**6**	508	34	5	37	21	22 (14)
**7**	2551	31	4	34	19	21 (13)
**8**	14670	19	2	20	8	14 (4)
**9**	92788	25	5	28	16	17 (10)
**Total**	**110668**	**171**	**35**	**182**	**100**	**122 (86)**

Table S1: RNAs in Nature identified by dual graphs in our motif library update, before adjustment with prior existing dual graphs.[1] For each vertex, the number of total graphs enumerated are shown. For current existing graphs, those found in the first, the second, and the combined search round are counted against vertex number, as well as those contain pseudoknots. For comparison, the total number of existing dual graphs in the prior library,[1] and those motifs common to both search protocols are listed.

Reason	Graph	PDB ID	Graph	PDB ID
**DNA-hybrid**	**5_6**	5F9R, 6MCB, 5Y36, 5B2T		
**Broken chains**	**5_11**	5U30	**6_153**	5FJC
	**6_369**	5FJC	**7_934**	3IZZ
	**7_1192**	4V92	**7_1235**	3JCS
	**7_2550**	5T5H	**8_721**	5GAN
	**8_4616**	4V92	**8_4760**	6AZ3
	**8_7922**	5ZWO	**8_12185**	5TC1
	**9_3051**	5YZG, 6EXN	**9_38598**	3J16
	**9_86359**	5GM6		
**Multiple chains**	**6_44**	4WQ1	**6_303**	2Z75
	**6_412**	6D6V	**7_581**	3JB9
	**7_628**	5LQW	**7_1949**	3G9C
	**9_4495**	5MPS	**9_20569**	5T2A
	**9_20790**	5T2A	**9_49214**	5JUP
**2D structure**	**5_71**	5C7W	**6_41**	6D9J
	**6_198**	5XY3	**6_251**	3HHN
	**7_2244**	3J2C	**8_5260**	4UYK
	**8_7933**	5IT9	**8_8221**	3J7O
	**8_8230**	2R8S	**8_14143**	4V8M

Table S2: Existing graphs found only in the prior study.[1] We group these graphs based on the reason that they are not found using the new search algorithm: interacting DNA chains were not included previously; broken chains were not separated previously; multiple interacting chains were not combined previously; 2D structure differences due to extraction program. For each graph, we list the PDB IDs of its corresponding RNA structures found previously.

Additional 2D Structure Screening

In our prior 2019 update,[1] 2D structures were extracted from RNA 3D structures using three programs: DSSR,[2] RNAView,[3] and MC-Annotate.[4] Only base pairs reported by at least two programs were taken into account as the "consensus" structure. Here, we choose DSSR as the main annotation program, because it provides more detailed structural analysis, such as identification of RNA/DNA chains and chain breaks. Though our dual graph representations are coarse-grained, occasional variations in the dual graph motifs do occur for some RNA structures.

To give an example, in Fig. S2, we plot the 2D structures obtained by the three programs for a ribonuclease P RNA (PDB ID: 3DHS), as well as the "consensus" structure derived in our prior study. While both DSSR and RNAView structures correspond to dual graph 9_38601, the "consensus" structure was found to have 10 helices and thus no dual graph assigned. This was due to base pair variations in Stem 1, i.e., an internal loop of 2-nt on both ends separates the stem into two.

For all RNAs assigned different dual graph motifs in our prior study,[1] we then perform additional 2D structure screening. We extract 2D structures using all three programs (DSSR, RNAView, and MC-Annotate), and identify corresponding dual graphs for each of them. The final motif is chosen as the one that has support from at least two programs. If the three programs give three different motifs, the one from DSSR is chosen. The screening results are listed in Table S3, and we re-assign motifs for only 4 RNAs.

PDB ID	Previous	Current	DSSR	RNAView	MC-Annotate
5C7W	5_71	4_27	4_27	4_27	4_27
6D9J	6_41	7_58	7_58	6_41	6_41
5XY3	6_198	7_1311	7_1311	6_198	6_198
3HHN	6_251	7_1341	7_1341	7_1341	7_1341
3J2C	7_2244	6_418	6_418	7_2244	4_4
4UYK	8_5260	9_19203	9_19203	9_19203	9_19203
5IT9	8_7933	9_38596	9_38596	8_7933	8_7933
3J7O	8_8221	7_1311	7_1311	8_8221	7_1311
2R8S	8_8230	9_20559	9_20559	9_20559	9_20559
4V8M	8_14143	>9 vertices	>9 vertices	>9 vertices	4_19
6D90	4_12	6_31	6_31	4_12	3_6
5ZWO	7_222	8_414	8_414	7_222	7_222
3DHS	>9 vertices	9_38601	9_38601	>9 vertices	9_38601
3P49	>9 vertices	9_87224	9_87224	>9 vertices	6_149

Table S3: Additional check for RNAs assigned different dual graphs in prior study[1] due to 2D structure extraction. For each RNA, we list the graph assigned previously and currently, and run three 2D extraction programs: DSSR,[2] RNAView,[3] and MC-Annotate.[4] The final dual graph motif is highlighted in yellow.

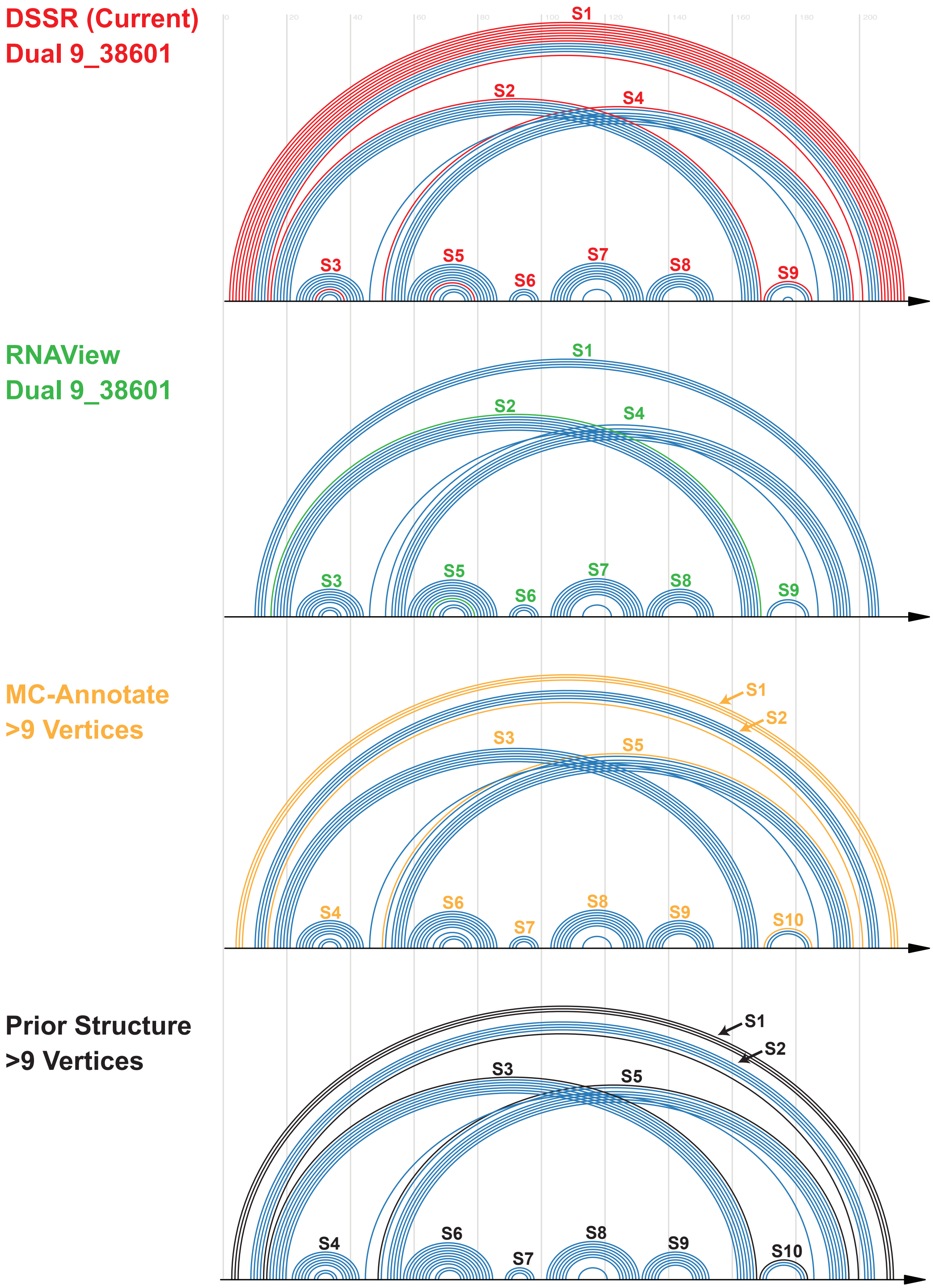

Figure S2: Arc plots of secondary structures extracted from a ribonuclease P RNA (PDB ID: 3DHS) using three programs. Common base pairs (or arcs) are drawn in blue, and those unique to DSSR/RNAView/MC-Annotate are drawn in red/green/yellow. The “consensus” structure derived in our prior study[1] is plotted at bottom, with common base pairs in blue and others in black.

Reason	Graph	PDB ID	Graph	PDB ID
**New RNA**	**4_22**	7D7W, 6TFF, 7D7V	**5_13**	7A5P
	**5_16**	7JNH, 7LJY	**5_50**	7ABG, 7AJU, 7NWG
	**5_67**	7K16	**5_79**	7A01, 6ZVK
	**6_78**	7AJU, 6ZQD	**6_123**	7DVQ
	**6_142**	6MCB	**6_232**	6VWL
	**6_245**	7LYG	**6_246**	6XMF
	**6_293**	7JJU	**6_302**	6UFM
	**6_309**	6ZDU	**6_396**	6QN3
	**7_52**	7MQA, 7MQ8	**7_119**	6ZQA
	**7_126**	6JQ5	**7_139**	6PMO
	**7_373**	6ZJ3	**7_718**	7LVA, 6QDV
	**7_979**	6MJ0	**7_1388**	6LQS
	**7_1693**	7LYF, 6DME	**7_1771**	7JRT
	**7_2245**	6UES	**8_2499**	7EAG
	**8_5390**	6ZYM	**8_7722**	6ZJ3
	**8_8010**	6Q97	**8_11622**	7ABG
	**8_12322**	7MQ8	**9_1477**	6QX9
	**9_3761**	6WDR	**9_16347**	7MQ8, 6LQP
	**9_20589**	6SXO	**9_24796**	6UFH, 6UFG
	**9_27699**	7AC7	**9_64993**	7SAM, 7SC6
**DNA-hybrid**	**3_7**	7OOP, 6XLJ, 5OIK, 6GML, 4BOC, 6JNX, 6GMH, 6FLP, 7MKO		
	**4_23**	6FLQ	**4_26**	5H9F
	**5_18**	6WBR	**5_39**	7EL1, 6M0X
	**6_71**	5B2T, 7OX9, 7OX8	**6_155**	5X2G
	**6_210**	5B2P	**6_253**	4V4B
	**6_418**	3JAN, 6R6P, 3J92, 3J2C	**6_493**	7BG9
	**6_496**	5U30	**7_31**	5WTI
	**7_189**	5F9R, 5Y36	**7_242**	6JDV
	**7_364**	7C7L	**7_1155**	6NY1, 6NY2
	**7_1812**	7LMA, 6D6V	**7_2393**	6VPC
	**7_2396**	1EGK	**8_4102**	7L49
**Broken chains**	**5_101**	6HA1, 5XYM, 6HTQ, 7A0S	**6_4**	5A8L
	**6_463**	1NJP, 4V49	**7_914**	6G90
	**8_3161**	2J37	**8_5518**	4V92
	**8_7791**	4D61		

Reason	Graph	PDB ID	Graph	PDB ID
Multiple chains	**5_43**	3JAP, 5OA3, 3J0P, 6Q97, 6YAL, 6O8W, 6HA8, 3J0O, 5TCU, 6XU7, 6FYY, 3J0L, 6YWE, 1ML5, 6WOO, 4V90, 7M4Y, 4KZZ, 7NWI, 7NHK, 7NHN, 6GAZ, 5ZLU		
	**4_7**	4V6E	**4_17**	2P7E
	**5_12**	1YLS	**5_76**	4K50
	**5_77**	6BK8, 5WSG	**6_120**	3J5Y
	**6_138**	1M5K	**6_166**	3J0L
	**8_2314**	3G9C, 2Z75	**8_2642**	5WSG
	**8_4305**	5Y88	**8_4787**	6ZJ3
	**8_5474**	7B9V, 6EXN	**9_8338**	4MGN
	**9_11105**	6EXN	**9_18869**	6G90
	**9_23630**	3JB9, 6ID1, 5YZG	**9_45828**	5LQW
	**9_51712**	5MPS, 5MQ0	**9_52640**	4W29
	**9_68582**	3J7R	**9_68604**	6AZ1
2D structure	**6_31**	6D90	**7_58**	6D9J
	**8_414**	5ZWO	**9_38601**	3DHS
	**9_87224**	3P49		

Table S4: Newly found existing graphs. We group these graphs based on the reason that they are not found in the prior study:[1] newly added PDB RNA structures after August 2018; interacting DNA chains were not included previously; broken chains were not separated previously; multiple interacting chains were not combined previously; 2D structure differences due to extraction program. For each graph, we list the PDB IDs of its corresponding RNA structures.

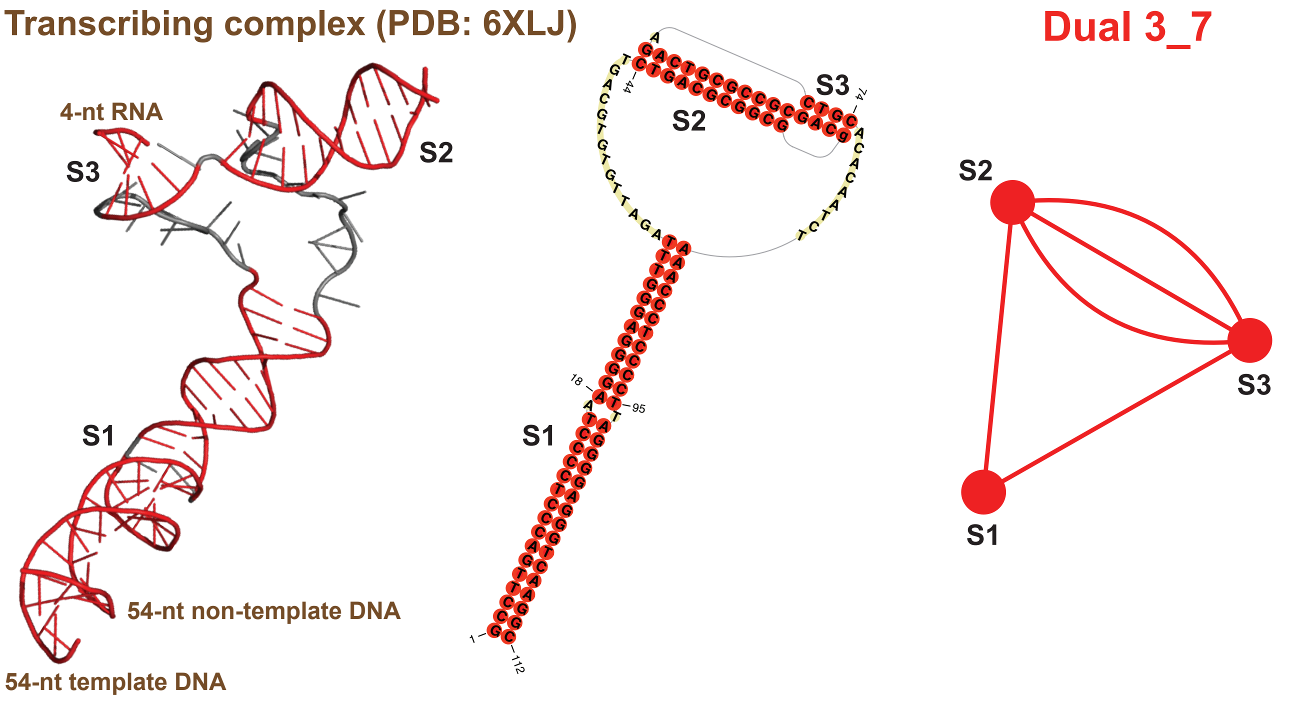

Figure S3: An example 3_7 structure. This transcribing complex (PDB ID: 6XLJ) consists of a 54-nt template DNA strand, a 54-nt complementary DNA strand, and a 4-nt transcribed RNA strand.

**Old existing dual graphs**			
**Graph**	**Type**	**Weight**	**Example RNA (PDB ID)**
8_3248	Multi RNA	2	Spliceosome postcatalytic P complex (6BK8)
9_18405	Single RNA	1	HIV-1 core packaging signal (2N1Q)
9_19203	Single RNA	1	Signal recognition particle RNA (4UYK)
9_20559	Single RNA	1	P4-P6 RNA ribozyme domain (2R8S)
9_21508	Single RNA	4	26S ribosomal RNA (6QKL)
9_35458	DNA hybrid	1	Ribosomal RNA expansion segment (3IZD)

**Newly found existing dual graphs**			
**Graph**	**Type**	**Weight**	**Example RNA (PDB ID)**
8_2314	Multi RNA	5	glmS ribozyme (3G9C)
8_2499	Multi RNA	1	Kink turns (7EAG)
8_3161	Single RNA	1	Ribosomal RNA (2J37)
8_4102	DNA hybrid	1	CRISPR-Cas12f complex (7L49)
8_4305	Multi RNA	1	Intron lariat spliceosome complex (5Y88)
8_12322	Single RNA	1	$5'$ external transcribed spacer ribosomal RNA (7MQ8)
9_11105	Multi RNA	1	Intron lariat spliceosome complex (6EXN)
9_18869	Multi RNA	1	Spliceosome complex (6G90)
9_27699	Single RNA	1	Transfer-messenger RNA (7AC7)

Table S5: Misclassified (non RNA-like) existing dual graph motifs using the Fiedler vector based clustering method in.[5]

References

[1] S. Jain, S. Saju, L. Petingi, and T. Schlick. An extended dual graph library and partitioning algorithm applicable to pseudoknotted RNA structures. *Methods*, 162-163:74–84, 2019.

[2] X. Lu and W. Olson. 3dna: a software package for the analysis, rebuilding and visualization of three-dimensional nucleic acid structures. *Nucleic Acids Res.*, 31:5108–5121, 2003.

[3] H. Yang, F. Jossinet, N. Leontis, L. Chen, J. Westbrook, H. Berman, and E. Westhof. Tools for the automatic identification and classification of RNA base pairs. *Nucleic Acids Res.*, 31:3450–3460, 2003.

[4] S. Lemieux and F. Major. RNA canonical and non-canonical base pairing types: a recognition method and complete repertoire. *Nucleic Acids Res.*, 30:4250–4263, 2002.

[5] Q. Zhu and T. Schlick. A fiedler vector scoring approach for novel RNA motif selection. *J. Phys. Chem. B*, 125:1144–1155, 2021.
